# Review of Pharmacological Properties and Chemical Constituents of *Pimpinella anisum*

**DOI:** 10.5402/2012/510795

**Published:** 2012-07-16

**Authors:** Asie Shojaii, Mehri Abdollahi Fard

**Affiliations:** Research Institute for Islamic and Complementary Medicine, Tehran University of Medical Sciences, Lalezar Street, Jomhuri Avenue, P.O. Box 1145847111, Tehran, Iran

## Abstract

*Pimpinella anisum (anise)*, belonging to Umbelliferae family, is an aromatic plant which has been used In Iranian traditional medicine (especially its fruits) as carminative, aromatic, disinfectant, and galactagogue. Because the wide traditional usage of *Pimpinella anisum* for treatment of diseases, in this review published scientific reports about the composition and pharmacological properties of this plant were collected with electronic literature search of GoogleScholar, PubMed, Sciencedirect, Scopus, and SID from 1970 to 2011. So far, different studies were performed on aniseeds and various properties such as antimicrobial, antifungal, antiviral, antioxidant, muscle relaxant, analgesic and anticonvulsant activity as well as different effects on gastrointestinal system have been reported of aniseeds. It can also reduce morphine dependence and has beneficial effects on dysmenorrhea and menopausal hot flashes in women. In diabetic patients, aniseeds showed hypoglycemic and hypolipidemic effect and reduce lipid peroxidation. The most important compounds of aniseeds essential oil were trans-anetole, estragole, *γ*-hymachalen, para-anisaldehyde and methyl cavicol. Due to broad spectrum of pharmacological effects, and very few clinical studies of *Pimpinella anisum*, more clinical trials are recommended to evaluate the beneficial effects of this plant in human models and synthesis of new drugs from the active ingredients of this plant in future.

## 1. Introduction

*Pimpinella anisum* L., a plant belonging to the Umbelliferae family, is one of the oldest medicinal plants. It is an annual grassy herb with 30–50 cm high, white flowers, and small green to yellow seeds, which grows in the Eastern Mediterranean Region, West Asia, the Middle East, Mexico, Egypt, and Spain [1]. *P. anisum* is primarily grown for its fruits (aniseeds) that harvested in August and September. Aniseeds contain 1.5–5% essential oil and used as flavouring, digestive, carminative, and relief of gastrointestinal spasms. Consumption of aniseed in lactating women increases milk and also reliefs their infants from gastrointestinal problems [2]. In the food industry, anise is used as flavoring and aromatic agent for fish products, ice cream, sweets, and gums [1, 3]. Despite the various studies which were conducted on *Pimpinella anisum*, there is no comprehensive review study on constituents and effectiveness of this plant. So the objective of this study was collecting all published articles about the chemical constituents and pharmacological properties of aniseeds with literature search of Googlescholar, PubMed, Sciencedirect, Scopus, and SID database from 1970 up to 2011.The agricultural studies and investigations on aniseeds tissue culture were excluded.

## 2. *Pimpinella anisum* in Traditional Medicine of Iran

Anise seeds are used as analgesic in migraine and also as carminative, aromatic, disinfectant, and diuretic in traditional medicine [4]. Aniseed has warm and dry nature and can increase milk production, menstruation, urine, and sweat secretion and also making good complexion. It is also effective in polishing of teeth. In some traditional texts, anise is mentioned for melancholy, nightmare, and also in treatment of epilepsy and seizure [5, 6].

## 3. Chemical Constituents

 Aniseed contains 1.5–6.0 mass % of a volatile oil consisting primarily of *trans*-anethole and also as much as 8–11 mass % of lipids rich in fatty acids, such as palmitic and oleic acids, as well as approximately 4 mass % of carbohydrates, and 18 mass % of protein [7]. Other studies have demonstrated the presence of eugenol *trans*-anethole, methylchavicol, anisaldehyde, estragole, coumarins, scopoletin, umbelliferone, estrols, terpene hydrocarbons, polyenes, and polyacetylenes as the major compounds of the essential oil of anise seed [8].Study of the essential oil of *Pimpinella anisum* L. fruits by GC and GC-MS showed the presence of *trans*-anethole (93.9%) and estragole (2.4%). Other compounds that were found with concentration higher than 0.06% were (E)-methyleugenol, **α**-cuparene, **α**-himachalene, **β**-bisabolene, *p*-anisaldehyde, and *cis*-anethole [3]. In another study for determination of the composition of essential oil of *Pimpinella anisum* L. fruits obtained from different geographical areas of Europe, in addition to the major components (*trans*-anethole (76.9–93.7%) and **γ**-himachalene (0.4–8.2%), some other compounds such as *trans*-pseudoisoeugenyl 2-methylbutyrate, *p*-anisaldehyde, and methylchavicol were also identified in essential oil [9]. Study of components of the whole plants and the seeds of *Pimpinella anisum* from Alberta showed that the major oil constituent (*trans*-anethole) was 57.4% of whole plant and 75.2% of seed oil. The other constituents of plant oil, present in amounts of 1–5% were *cis*-anethole, carvone, **β**-caryophyllene, dihydrocarvyl acetate, estragole and limonene [10].

The chemical constituents of aniseed extract obtained by Supercritical extraction using CO_2_ were determined by GC-MS. The major compounds were anethole (~90%), **γ**-himachalene (2−4%), *p*-anisaldehyde (<1%), methylchavicol (0.9−1.5%), *cis*-pseudoisoeugenyl 2-methylbutyrate (~3%), and *trans*-pseudoisoeugenyl 2-methylbutyrate (~1.3%) [11]. A new terpene hydrocarbon called neophytadiene was isolated from aniseed in 1978 [12]. 4-(**β**-d *-*glucopyranosyloxy) benzoic acid which is one of the phenolic glycosides of the Umbelliferae family was also isolated from aniseed [13]. In a study by Fujimato et al., four aromatic compound glucosides, an alkyl glucoside, and a glucide were isolated as new compounds from the polar portion of methanolic extract of anise fruits. The structures of the new compounds were clarified as (E)-3-hydroxy-anethole **β**-d-glucopyranoside, (E)-10-(2-hydroxy-5-methoxyphenyl) propane **β**-d-glucopyranoside, 3-hydroxyestragole **β**-d-glucopyranoside, methyl syringate 4*-O-*β**-d-glucopyranoside, hexane-1,5-diol 1-*O-*β**-d-glucopyranoside, and 1-deoxy-l-erythritol 3-*O*-**β**-d-glucopyranoside [14]. Isolation and structure elucidation of flavonoid constituents from anise, caraway, coriander, and fennel by means of chromatography on cellulose columns lead to isolation of quercetin 3-glucuronide, rutin, luteolin 7-glucoside, isoorientin, and isovitexin as crystalline compounds and apigenin 7-glucoside and a luteolin glycoside as noncrystalline compounds from anise [15].

In another study, a silver ion HPLC procedure was used to determine the fatty acids composition of aniseed oil. The results showed the positionally isomeric 18:1 fatty acids oleic acid (*cis* 9–18:1), petroselinic acid (*cis* 6–18:1), and *cis*-vaccenic acid (*cis* 11–18:1), in aniseed oil by a single gradient run on a single cation exchange column laboratory converted to the silver ion form [16]. Also three lignin-carbohydrate-protein complexes were isolated from a hot water extract of the seeds of *Pimpinella anisum* by combination of anion-exchange, gel filtration, and hydrophobic interaction column chromatographies [17].

## 4. Pharmacological Properties (Table 1)

### 4.1. Antibacterial and Antifungal Effects

The antibacterial activities of the aqueous, 50% (v/v) methanol, acetone and petroleum ether extracts of *Pimpinella anisum* L. fruits were tested against 4 pathogenic bacteria (*Staphylococcus aureus, Streptococcus pyogenes, Escherchia coli, and Klebsiella pneumoniae*) by disc diffusion method. The results showed that only aqueous and methanol extracts exhibited fair antibacterial activity against all of the test bacteria and the aqueous extract was found to be more effective than methanolic extract, whereas acetone and petroleum ether extracts cannot inhibit the growth of the pathogenic test bacteria [18].

Antimicrobial effects of water and ethanolic extracts of aniseed were studied by Gulcin et al. against 10 bacterial species and *also Candida albicans* with disc diffusion method. In this study, ethanolic extract showed significant inhibitory activity against all tested bacteria but not effective on *Candida albicans*. However, the antimicrobial effect of water extract was not detected against Gram-negative bacteria, *Pseudomonas aeruginosa*, and *Escherichia coli*, but it was effective against *Candida albicans* [8]. The alcoholic extracts of *Pimpinella anisum* seeds also showed antibacterial activity against *Micrococcus luteus* and *Mycobacterium smegmatis* [19].

In another study, synergic antibacterial activity between *Thymus vulgaris* and *Pimpinella anisum* essential oil and methanol extract was evaluated against 9 pathogenic bacteria. Essential oil and methanol extract of these plants exhibited antibacterial activity against most tested pathogens, and the maximum effect was observed against *Staphylococcus aureus*, *Bacillus cereus,* and *Proteus vulgaris*. However, combination of essential oil and methanol extracts of these plants showed an additive effect against most tested bacteria especially *Pseudomonas aeruginosa* [20]. The antibacterial potential of aqueous decoctions of black pepper, bay leaf, aniseed, and coriander against 176 bacterial isolates belonging to 12 different genera were detected by the mean of disc diffusion technique. The findings showed that the aqueous decoction of black pepper was the most bacterial-toxic exhibited 75% antibacterial activity and decoction of aniseed exhibited 18.1% antibacterial activity (maximum antibacterial activities exhibited against *Micrococcus roseus*) [21]. In addition to antibacterial activity, the essential oil of aniseed showed significant inhibitory activity against fungi, and the most active component of it was anethol [22]. In a study by Kosalec et al., antifungal activities of fluid extract and essential oil of anise fruits studied on seven species of yeasts and four species of dermatophytes by the means of diffusion method with cylinders and the broth dilution method. The findings revealed that the fluid extract of aniseed showed antimycotic activity against *Candida albicans*, *C. parapsilosis*, *C. tropicalis*, *C. pseudotropicalis,* and *C. krusei,* and the largest inhibition zone was observed in *C. albicans*. It is also showed inhibitory effect against dermatophyte species (*Trichophyton rubrum, T. mentagrophytes*, *Microsporum canis,* and *M. gypseum*), and the largest inhibitory zone was detected for *T. mentagrophytes*. The essential oil of anise exhibited strong antifungal activity against yeasts and dermatophytes, and the largest inhibition zone was found in *C. parapsilosis* (30 mm), followed by *C. albicans*, *C. glabrata,* and *Geotrichum *spp. In this study, anise essential oil exhibited stronger antifungal activities rather than its extract against yeasts and dermatophytes [23]. Antifungal activity of anise essential oil also was reported against *Aternaria alternata*, *Aspergillus niger,* and *Aspergillus parasiticus,* and the most effected fungus was *A. parasiticus* [3]. Investigation of antifungal activity of methanolic extracts of aniseed against four dermatophyte species, and one saprophyte fungus showed that the extract inhibited only dermatophyte species included *Candida albicans*, *Trichophyton mentagrophyte*, and *Microsporum canis* but demonstrated no inhibitory effect on saprophyte *A. niger* [24].

### 4.2. Muscle Relaxant Effect

The relaxant effect of *Pimpinella anisum *on isolated guinea pig tracheal chains and its possible mechanism was studied by Boskabady and Ramazani-Assari. In this research, the bronchodilatory effects of aqueous and ethanol extracts and essential oil of anise were examined on precontracted isolated tracheal chains of the guinea pig by 10 mM methacholine in two different conditions including nonincubated tissues (group 1) and incubated tissues with 1 mM propranolol and 1 mM chlorpheniramine (group 2).The results showed that aqueous and ethanol extracts, essential oil, and theophylline (1 mM) showed significant relaxant effects compared to those of controls. The relaxant effects of aqueous and ethanol extracts were not significantly different from that of theophylline, but the effect of essential oil was significantly lower than theophylline. There was also no significant difference between the relaxant effects obtained in group 1 and 2 experiments. The results also showed that the relaxant effect of this plant is due to inhibitory effects on muscarinic receptors [32].

In another study, antispasmodic and relaxant effects of three hydroalcoholic extracts of the aerial parts of *Pimpinella anisum* (ethanol : water; 40 : 60, 60 : 40, and 80 : 20) were investigated on rat anococcygeus smooth muscle. The entire three hydroalcoholic extracts attenuated acetylcholine-induced contraction. The finding of this study described that only the extract contains 60% ethanol (HA60) showed concentration dependently relaxed acetylcholine-precontracted tissues, but two other hydro alcoholic extracts cannot produce relaxation. Studying the possible mechanisms underlying the relaxant effect showed that this effect is mainly dependent on the activation of the NO-cGMP pathway [33].

### 4.3. Anticonvulsant Effect

 Anticonvulsant effects of an essential oil of the fruits of *Pimpinella anisum *were studied against seizures induced by pentylenetetrazole (PTZ) or maximal electroshock (MES) in male mice. This study revealed that *P*. *anisum *increases the threshold of clonic seizures induced by i.v. infusion of PTZ, and it can also block tonic convulsions induced by i.p. injection of PTZ. Moreover, *P*. *anisum *possesses anticonvulsant activity against tonic seizures induced by MES [34]. In another study by Heidari and Ayeli, the effect of methyl alcoholic extract of anise on picrotoxin-induced seizure in mice was studied. The results showed that anise extract caused an increased delay in the onset of seizure in the mice which had been pretreated with different doses of the extract, and the most effective dose was 200 mg/kg (*P* < 0.05). In addition, this dose delayed the time of death in mice (*P* < 0.01) more satisfactory than phenobarbital (40 mg/kg) on delaying death time [35]. Results of the other study for investigating the effect of the aqueous extracts of leaves and stems of some Arab medicinal plants including* Pimpinella anisum* on the picrotoxin-induced seizures in mice revealed that the extracts of *Rosmarinus officinalis, Pimpinella anisum, Matricaria chamomilla,* and *Artemisia vulgaris* were found to delay the onset of picrotoxin-induced seizures and to decrease the mortality rate [36].

In year 2008, Janahmadi et al. studied the cellular mechanisms of antiseizure effect of anise fruits. In this study, they determined whether the fruit essential oil of anise affect the bioelectrical activity of snail neurons in control condition or after pentylenetetrazol-(PTZ-) induced epileptic activity. The results indicate that the candidate cellular mechanisms probably underlying the hyperexcitability produced by anise oil include enhancement of Ca^2+^ channels activity or inhibition of voltage and/or Ca^2+^ dependent K^+^ channels activity underlying post-hyperpolarization potential [54].

### 4.4. Effect on Gastrointestinal System

#### 4.4.1. Effect on Gastric Ulcer

For studying the effect of aqueous suspension of anise against gastric ulcers in rat, acute gastric ulceration was produced by various noxious chemicals and indomethacin. The results showed that anise significantly inhibited gastric mucosal damage induced by necrotizing agents and indomethacin. The antiulcer effect was further confirmed histologically [41].

#### 4.4.2. Palliation of Nausea

In a case study, an aromatherapy treatment containing *Pimpinella anisum*, *Foeniculum vulgare,* var. *dulce*, *Anthemis nobilis,* and *Mentha piperita* was examined in twenty-five patients suffering from the symptoms of nausea in a hospice and palliative care program. A majority of patients who used the aromatherapy treatments reported relief. However, all patients in this study were also using a variety of other treatments for their symptoms [42].

#### 4.4.3. Effect on Constipation

The laxative efficacy of a phytotherapic compound containing *Pimpinella anisum* L., *Foeniculum vulgare* Miller, *Sambucus nigra* L., and *Cassia augustifolia* was studied in a randomized clinical trial included 20 patients presenting with chronic constipation according to the criteria of the American Association of Gastroenterology. The primary endpoint was colonic transit time (CTT), measured radiologically. Secondary endpoints included number of evacuations per day, perception of bowel function, adverse effects, and quality of life. The results of the study revealed significant laxative effects of phytotherapic compound when compared with placebo. This effect was demonstrated by a decrease in colonic transit time as well as an increase in the number of daily evacuations. Although quality of life did not show significant differences among the study periods and no significant differences were observed in terms of adverse effects throughout the study period, so this compound can be a safe alternative option for the treatment of constipation [43].

### 4.5. Effect on Morphine Dependence

The effects of essential oil of *Pimpinella anisum* on the expression and acquisition of conditioned place preference (CPP) induced by morphine in mice were studied. The findings showed that subcutaneous injections of morphine (2–5 mg/kg) produced place preference in a dose-dependent manner and injection of essential oil of *P. anisum* may induce conditioned place aversion in mice, that is, the essential oil has some aversive effects as investigated by place conditioning paradigm. In addition, this oil has also a GABA ergic effect [40].

### 4.6. Analgesic and Anti-Inflammatory Effect

Screening of some Iraqi medicinal plants for analgesic activity showed that the extracts of *Tribulus terrestris* and *Pimpinella anisum* exhibited significant analgesic activity versus benzoquinone-induced writing and in thermal tests [37]. In a study by Tas, essential oil of *Pimpinella anisum* showed significant analgesic effect similar to morphine and aspirin [38]. Also fixed oil of anise was investigated for anti-inflammatory and analgesic activity in mice. The finding showed that the fixed oil of anise has anti-inflammatory effect as strong as indomethacin and it showed analgesic effect comparable to that of 100 mg/kg aspirin and 10 mg/kg morphine at 30th min [39].

### 4.7. Effect on Menopausal Hot Flashes

In a double blind clinical trial, the effect of anise extract on menopausal hot flashes in 72 postmenopausal women was examined. In this study, consumption of 3 capsules of anise extract (each capsule contains 100 mg of extract) for 4 weeks leads to significant reduction in hot flash frequency and intensity and in postmenopausal women [51].

### 4.8. Effect on Dysmenorrhea

In a study by Khoda Karami et al., the effectiveness of a herbal capsule containing dried extracts of celery, saffron, and anise was compared with mefenamic acid capsule in 180 female students (with age 17–28) with primary dysmenorrhea. The results showed significant reduction in pain intensity in both herbal and mephnamic acid group compare to placebo group. Also the results revealed that the effectiveness of herbal capsule was better than mephnamic acid in pain relief and can be a suitable alternative in primary dysmenorrhea [53].

### 4.9. Antioxidant Activity

 In a study by Gulcin et al., the antioxidant properties of water and ethanolic extracts of aniseeds were evaluated using different antioxidant tests, and antioxidant activities were compared with synthetic antioxidants such as butylated hydroxyanisole (BHA), butylated hydroxytoluene (BHT), and *α*-tocopherol. Both extracts of aniseeds showed strong antioxidant activity, reducing power, DPPH radical and superoxide anion scavenging, hydrogen peroxide scavenging, and metal chelating activities compared to BHA, BHT, and *α*-tocopherol, and water extract exhibited greater antioxidant capacity than ethanolic extract [8].

Also investigation of in vitro and in vivo antioxidant potential of aniseeds showed that ethanolic extract of aniseed displayed scavenging activity against nitric oxide, superoxide and 1,1-diphenyl, 2-picryl hydrazyl (DPPH) radicals and reducing power in a concentration-dependent manner [45].

The antioxidant potential of essential oil and oleoresins from anise seeds was studied. The antioxidant activities were assessed by inhibition of linoleic acid peroxidation, 1,1-diphenyl-2-picrylhydrazyl (DPPH) radical scavenging, Fe^3+^ reducing power, and various lipid peroxidation assays. The findings showed that the anise oil and its methanol oleoresin showed highest antioxidant activity, even higher than BHA and BHT. However, the antioxidant activities of other oleoresins were somewhat lower [48].

Screening of antioxidant properties of some Umbelliferae fruits from Iran (including* Pimpinella anisum*) by the DPPH (2,2′-diphenyl-1-picrylhydrazyl) radical scavenging test showed that all the extracts exhibited antioxidant capability, and *P. anisum *extract (IC_50_ = 109.80) exhibited the strongest activity and the ethyl acetate fraction of the extract exhibited the highest activity and flavonoid content. A positive correlation was found between the antioxidant potency and flavonoid content of the fractions [49].In another study the antioxidant activity of water and alcohol extracts of chamomile flowers, anise seeds, and dill seeds was investigated. The extracts showed marked antioxidant activity in both linoleic acid and liposome model systems, although the water extracts showed higher antioxidant activity than the corresponding alcohol extracts. Also antioxidant activity of aniseeds was lower than chamomile flowers and dill seeds [47].

The antioxidant properties of thirteen herbal teas including anise tea was assessed in vitro. The finding indicate that anise tea showed weak antioxidant activity in Trolox-equivalent antioxidant capacity (TEAC assay) and moderate effect in hypochlorite quenching assay and peroxynitrite quenching assay rather than other herbal teas [50].

### 4.10. Insecticidal Effects

Plant essential oils from 40 plant species including *Pimpinella anisum* were tested for their insecticidal activities against larvae of *Lycoriella ingenua* using a fumigation bioassay. Some of the essential oil including anise and garlic essential oil showed good insecticidal activity against the larvae. Among the identified compound in effective essential oils, allyl isothiocyanate was the most toxic against larvae of *L. ingenua* followed by *trans*-anethole, diallyl disulfide, and *p*-anisaldehyde [25].

Prajapati et al. showed that the essential oils of *Juniperus macropoda* and *Pimpinella anisum* were highly effective as larvicidal and ovicidal against three mosquito species [26]. Also the anise essential oil showed repellency against mosquito *Culex pipiens* [27]. In another study, the exposure to vapours of essential oils from anise and cumin resulted in 100% mortality of the eggs of two stored-product insects (the confused flour beetle, *Tribolium confusum*, and the Mediterranean flour moth, *Ephestia kuehniella*) [28].

The acaricidal activity of *p*-anisaldehyde derived from anise seed oil and commercially available components of anise seed oil were studied against the house dust mites, *Dermatophagoides farina,* and *D. pteronyssinus*. The results showed that the compound most toxic to these dermatophagoides were *p*-anisaldehyde followed by benzyl benzoate and, therefore, *p*-anisaldehyde may be useful as a lead compound for the selective control of house dust mites [29].

### 4.11. Antiviral Effects

The effects of the essential oil of *Foeniculum vulgar* and *Pimpinella anisum* were examined against PVX (potato virus), TMV (tobacco mosaic virus) and TRSV (tobacco ring spot virus), on the hypersensitive host *Chenopodium amaranticolor*. The essential oil is at 3000 ppm completely inhibited PVX, TMV, and TRSV [30].

Three lignin-carbohydrate-protein complexes (LC1, LC2, and LC3) with antiviral and immunostimulating activity were isolated from a hot water extract of seeds of *Pimpinella anisum* by combination of anion exchange, gel filtration, and hydrophobic interaction column chromatographies. These complexes showed antiviral activities against herpes simplex virus types 1 and 2, human cytomegalo virus, and measles virus. Also the effects of these complexes in activation of macrophage were investigated. After RAW 264.7, murine macrophage cells had been incubated with these compounds for 20 h, and the nitric oxide (NO) production was enhanced in dose-dependent manner. The effects of these compounds on mRNA and protein expression of inducible nitric oxide synthase (iNOS) in RAW 264.7 cells showed that they induced mRNA iNOS expression in a time-dependent manner. Furthermore, they induced expression of both IL-1*β* and IL-10 mRNAs. These results suggest that the lignin-carbohydrate-protein complexes from *P. anisum* possessed potency as functional food ingredients against infectious diseases [31].

### 4.12. Effects on Diabetic Patients

The antidiabetic, hypolipidemic, and antioxidant activities of aniseeds and coriander seeds were compared in type 2 diabetic patients. The seed powders (5 g/day) were administered to two groups of type 2 diabetes patients for 60 days. The results indicated 11% rise of fasting blood glucose in control and 36% decrease in aniseed-treated, and 13% decrease in coriander-treated type 2 diabetics. Also significant decrease in serum cholesterol and triglycerides in aniseed treated and coriander seed-treated patient was observed. Protein oxidation in serum and lipid peroxidation in erythrocytes and plasma was decreased in both treated groups as compared with the initial values. Both the groups showed rise in serum *β*-carotene and vitamin A levels which could have resulted in a significant decrease in lipid peroxidation in RBC and plasma, and also rise in vitamin C was detected in both anise and coriander group. So both the seeds have antidiabetic, hypolipidemic and antioxidant effects in diabetic patient [45, 46].

### 4.13. Effect on Glucose Absorption

The effect of aniseed oil on the absorption of glucose from the jejunum and water from the colon and kidney tubules and also its mechanism of action were studied by Kreydiyyeh et al. The findings showed that aniseed oil increased significantly glucose absorption in the rat jejunum, because the oil enhanced the activity of the Na^+^-K^+^ ATPase which increases the sodium gradient that gears the mucosal glucose transport. However, adding of anise oil at a 0.05% concentration did not have any significant effect on colonic water absorption, and it seems thus that the ATPase in the colon is resistant to the oil effect. Furthermore, adding aniseed oil to drinking water, reduced the volume of urine produced in the rat and increased the activity of the renal Na^+^-K^+^ ATPase even at extremely low concentrations [44].

### 4.14. Effect on Broiler Performance

In the study by Ciftci et al., adding 400 mg/kg of anise oil to the diet of day-old broilers was improved feed conversion ratio by approximately 6% compared to antibiotic group. So anise oil can be considered as a natural growth promoter for poultry [52].

### 4.15. Effect on Milk Production

The effects of diet supplementation with aniseed and fenugreek seeds on the performance does and kits were studied.Finding revealed that the daily milk intake of kits in anisum-fenugreek group was equivalent to that of control rabbits. Also, the 17 days body weight did not differ significantly between two groups. At 35 days of lactation, the differences between anisum-fenugreek group and control groups were not significant in litter size, litter weight, kit weight and 1–35 day weight, gain. In conclusion, further studies are needed to investigate the palatability and optimal level of these spices in the feed of lactating rabbits [55].

## 5. Discussion

Plants have been the origins of many drugs from the ancient times. Pharmacological evaluations of plants may be due to new natural agents for treatment of disease. Furthermore, identification of the active principles of these medicinal plants has an important role in introducing new drugs.

*Pimpinella anisum* is one of the medicinal plants which have been used for different purposes in traditional medicine of Iran. So far, different studies were performed on the extracts and essential oil of *Pimpinella anisum* to identify the chemical compounds and pharmacological properties of this plant, and various properties such as antimicrobial, antifungal, antiviral, antioxidant, and insecticidal effects have been reported of aniseeds. The findings also revealed that aniseeds can cause gastric protection, muscle relaxant, and affect digestive system. In diabetic patients, it has hypoglycemic and hypolipidemic effects and reduces lipid peroxidation. Furthermore, aniseeds showed anticonvulsant effect, reduced morphine dependence, and induced conditioned place aversion in mice. Aniseed also has beneficial effects on dysmenorrhea and menopausal hot flashes in women. The most important compounds of aniseeds essential oil were *trans*-anethole, estragole, *γ*-hymachalen, *p*-anisaldehyde, and methyl chavicol. Due to broad spectrum of pharmacological effects of this plant, and very few clinical studies performed on this plant, more clinical trials are recommended to evaluate the beneficial effects of *Pimpinella anisum* in human models and identification of active compounds of this plant which can lead to synthesis of new drugs from the active ingredients in future.

## Figures and Tables

**Table 1:** The pharmacological effects of *Pimpinella anisum*.

System	Effect	Preparation	Reference
Organism	Antibacterial	Aqueous and 50% (v/v) methanol extract	[18]
		Ethanol extract	[8, 19]
		Essential oil and methanol extract (in combination with *Thymus vulgaris*)	[20]
		Aqueous decoction	[21]
	Antifungal	Essential oil	[3, 22]
		Fluid extract	[23]
		Methanol extract	[24]
	Insecticidal	Essential oil	[25–29]
		*p*-Anisaldehyde from aniseed oil	[29]
	Antiviral	Essential oil	[30]
		Lignin-carbohydrate-protein complexes from hot water extract	[31]

Muscle	Muscle relaxant of tracheal chain	Aqueous extract	[32]
		Ethanolic extract	[32]
		Essential oil	
	Antispasmodic and relaxant of anococcygeus smooth muscle	Hydroalcoholic extract (60% ethanol)	[33]

		Essential oil	[34]
	Anticonvulsant	Methanol extract of seeds	[35]
		Aqueous extract of leaves and stem	[36]
nervous system		extract	[37]
	Analgesic	Essential oil	[38]
		Fixed oil	[39]
	Conditioned place aversion in morphine dependence	Essential oil	[40]

	Antiulcer	Aqueous suspension	[41]
	Palliation of nausea	Essential oils of aniseeds, *foeniculum vulgar, Anthemis nobilis, *and *Mentha piperita *	[42]
Gastrointestinal	Laxative	Phytotherapic compound of anise and *foeniculum vulgar, Sambucus nigra, Cassia angustifolia *	[43]
	Increase glucose absorption from the jejunum	Essential oil	[44]

Renal	reduce volume of urine by increase activity of the renal Na^+^-K^+^-ATPase	Essential oil	[44]

Endocrine	Antidiabetic	Seed powder	[45, 46]
	Hypolipidemic		

Immune system	Antioxidant	Ethanol extract	[8, 45, 47]
		Water extract	[8, 47]
		Essential oil	[48]
		Oleoresin	[48]
		Ethyl acetate fraction ofethanol extract	[49]
		Anise tea	[50]
	Increase of *β*-carotene, vitamins A, C	Seed powder	[46]

	Reduction of menopausal hot flashes	Capsules of anise extract	[51]
Others	Growth promoter of day-old broilers	Essential oil	[52]
	Reduction of pain in dysmenorrhea	Herbal capsule (extracts of anise, celery, saffron)	[53]
